# Kinetic Analysis of the Motility of Giant Virus-Infected Amoebae Using Phase-Contrast Microscopic Images

**DOI:** 10.3389/fmicb.2019.03014

**Published:** 2020-01-17

**Authors:** Sho Fukaya, Keita Aoki, Mio Kobayashi, Masaharu Takemura

**Affiliations:** ^1^Laboratory of Biology Education, Department of Mathematics and Science Education, Graduate School of Science, Tokyo University of Science, Tokyo, Japan; ^2^Laboratory of Biology, Department of Liberal Arts, Faculty of Science, Tokyo University of Science, Tokyo, Japan

**Keywords:** kinetic analysis, *Acanthamoeba*, giant virus, phase-contrast microscopic image, time-lapse, image analysis

## Abstract

Tracking cell motility is a useful tool for the study of cell physiology and microbiology. Although phase-contrast microscopy is commonly used, the existence of optical artifacts called “halo” and “shade-off” have inhibited image analysis of moving cells. Here we show kinetic image analysis of *Acanthamoeba* motility using a newly developed computer program named “Phase-contrast-based Kinetic Analysis Algorithm for Amoebae (PKA3),” which revealed giant-virus-infected amoebae-specific motilities and aggregation profiles using time-lapse phase-contrast microscopic images. This program quantitatively detected the time-dependent, sequential changes in cellular number, size, shape, and direction and distance of cell motility. This method expands the potential of kinetic analysis of cultured cells using versatile phase-contrast images. Furthermore, this program could be a useful tool for investigating detailed kinetic mechanisms of cell motility, not only in virus-infected amoebae but also in other cells, including cancer cells, immune response cells, and neurons.

## Introduction

Automated cell tracking is a useful tool for the discovery of hidden biological insights in large datasets and the elucidation of physiological and microbiological processes (Gilbert et al., 2010; Masuzzo et al., 2016; Ker et al., 2018). In multicellular organisms, the migration of cells plays a fundamental role in various cellular processes, including the immune response, formation of the neural network, and cancer metastasis. Thus, algorithms have been reported that track muscle stem cells automatically or manually and reveal cell lineage relationships (Gilbert et al., 2010; Ker et al., 2018). Additionally, in unicellular organisms, cell motilities reveal their behaviors in their own populations and ecosystems (Mathijssen et al., 2019). The quantitative analysis of these cell motilities could have merit in future studies of such various cellular processes, and the behavioral interaction between cells and inorganics, cells and cells, or cells and viruses. When analyzing the kinetics of living cells, phase-contrast microscopy can be used to avoid labeling of the cell content, which can affect cells and analysis results. When observing the cells with the phase-contrast microscope, artifacts called “halo,” in which the cells are brightly surrounded, and “shade-off,” in which the intensity inside the cells is similar to the background, are found. These artifacts can hinder the detection of cells when using typical image analysis algorithms; so, fluorescence or quantitative phase-contrast microscopy are generally used to detect cells (Yin et al., 2012; Handala et al., 2019; Ozaki et al., 2019). Recently, amoeba cell morphological analysis using phase-contrast microscopic images has been reported (Wandro, 2017). An image analysis method of phase-contrast microscopic movies for quantification of intracellular motions has also been reported (Osváth et al., 2018). It should be possible to develop an image analysis method for phase-contrast microscopic images specialized for cell motilities, which will broaden the scope for the study of cell migration and motilities.

*Acanthamoeba*, one of the abundant free-living protozoans, is a known pathogenic microorganism that causes *Acanthamoeba* keratitis (Khan, 2015). It is a known laboratory host of so-called “giant viruses,” including the families *Mimiviridae* and *Marseilleviridae*, pandoraviruses, pithoviruses, molliviruses, and medusavirus (La Scola et al., 2003; Boyer et al., 2009; Abergel et al., 2015; Yoshikawa et al., 2019). After infection with a giant virus, *Acanthamoeba* cells show various morphological and behavioral changes, termed cytopathic effects (CPE), and are then killed. Recent studies have indicated that amoeba cells infected with giant viruses show various CPEs, such as cell-rounding, encystment, lysis, and formation of cell aggregates called “bunches” (Reteno et al., 2015; Aoki et al., 2019; Oliveira et al., 2019; Yoshikawa et al., 2019). Among these CPEs, cell-rounding or lysis are the common CPEs to check and confirm the infection of amoeba with giant viruses. However, no quantitative studies have been reported as to which kinetics cause these transformations and what happens in amoeba cells from the onset of viral infection to their death, from a cellular ethological point of view. The detailed behavioral changes of amoeba populations, from the viewpoint of “hosts” of giant viruses, are not well-understood.

Recently, we isolated new members of the family *Marseilleviridae*, kyotovirus, kashiwazakivirus, and hokutovirus from Japan (Aoki et al., 2019). Kyotovirus causes cell-rounding in infected amoeba, as previously reported in other members of the family *Marseilleviridae* (Boyer et al., 2009; Dos Santos et al., 2016). On the other hand, kashiwazakivirus and hokutovirus lead infected amoeba cells to form “bunches,” as previously reported in tupanviruses (Oliveira et al., 2019). It is not yet understood how “bunches” form after kashiwazaki- and hokutovirus infections, from both amoebal ethological and viral aspects. To analyze the process of infection with giant viruses, the alteration of amoeba populations needs to be understood. To reveal the process of infection, we developed a new, specialized kinetic analytical program for tracing each amoeba cell in an amoeba population from time-lapse phase-contrast microscopic images, named “Phase-contrast-based Kinetic Analysis Algorithm for Amoebae (PKA3),” which is useful for the quantification of cellular numbers, size, shape, and the direction and distance of cell motility.

## Methods

### Amoebae and Viruses

*Acanthamoeba castellanii* (Douglas) Neff (ATCC 30010^TM^) cells were cultured in PYG (proteose-peptone-yeast extract-glucose) medium as previously described (Aoki et al., 2019; Yoshikawa et al., 2019). Members of the family *Marseilleviridae*, hokutovirus 1 and kyotovirus 1 (Aoki et al., 2019), that were isolated in our laboratory from water samples from Japan were used in this study. Virus titers and the multiplicity of infection (M.O.I.) were calculated as previously described (Aoki et al., 2019).

### Time-Lapse Images

One hundred microliters of *A. castellanii* cells in PYG medium at a concentration of 20,000 cells/μL were grown in each well of a 96-well microplate, followed by infection with the viruses (M.O.I. = 1). Time-lapse images of control *A. castellanii* cells and virus-infected *A. castellanii* cells were obtained using all-in-one fluorescent microscopy BZ-X800/X810 (Keyence Co., Osaka, Japan) with a 4x objective lens (CFI Plan Fluor DL 4x, NIKON INSTECH CO., LTD. Tokyo, Japan). This microscope allows time-lapse microscopic imaging in bright field, phase-contrast, and fluorescence modes. In addition, images of multiple wells of a microplate can be obtained in parallel. We did not use fluorescence microscopy in this study, but used this microscope system instead to simultaneously process multiple samples. A total of 2,880 phase-contrast images were obtained from each well by taking every 45 s for 36 h.

### Development of the Program

Time-lapse images of a phase-contrast microscopic images without special customization for these purposes were used for analysis. The analysis was performed using a self-written C/C++ program. To speed up the analysis process, an OpenCL heterogeneous compute framework on a graphics processing unit was used (The Khronos OpenCL Working Group, 2011). A Surface Laptop 2 computer (Microsoft Co., WA, USA) with CPU (Core i5-8250U, Intel Co., CA, USA), 8.0 GB memory, and embedded GPU (UHD Graphics 620, Intel Co., CA, USA) was used to develop the program. A Precision 3430 (Dell Inc., TX, USA) desktop computer with CPU (Xeon E-2124, Intel Co., CA, USA), 16.0 GB memory, and GPU (Quadro P400, NVIDIA, CA, USA) was used to operate the microscope and analyze the images. With this setup, an average of ~150 ms was required to analyze each 960 × 720 pixels image of this study. The source code of PKA3 is available from the corresponding authors on reasonable request.

### Statistical Analysis

Statistical significance of each result was analyzed using Mann–Whitney U two-sided test. The *n* and *p*-values are shown in each figure.

## Results

### Development of Analytical Method

**Figure 1:**
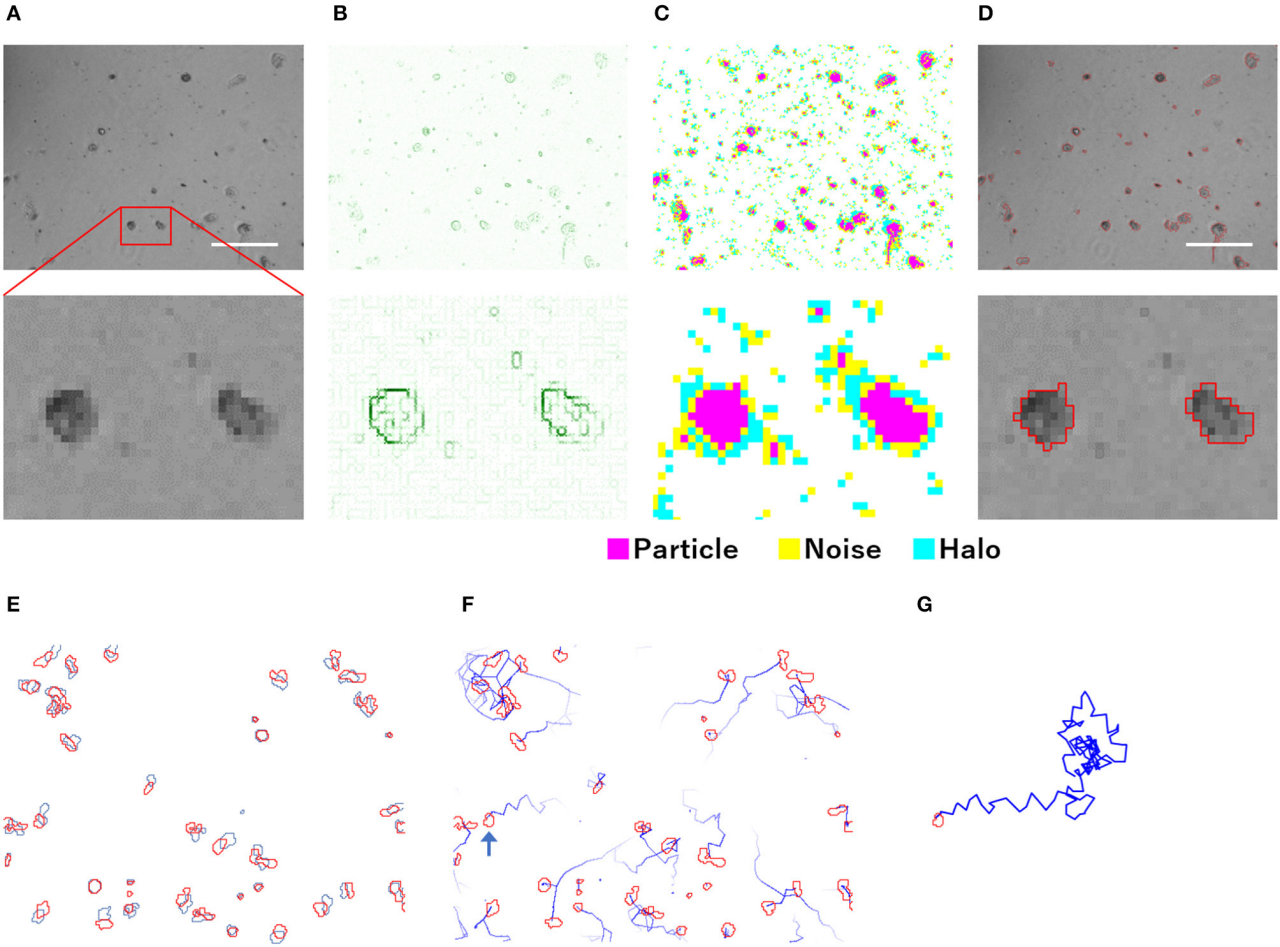
Overview of the analysis of phase-contrast microscopic images by PKA3. **(A)** Phase-contrast microscopic images of non-infected *A. castellanii*. **(B)** Edges detected based on the intensity gradient of the phase-contrast in **(A)**. **(C)** Pixels automatically identified based on edges and intensity data. **(D)** Analyzed particles are outlined in red. **(E)** Locomotion of each particle from the first frame (red) to the next frame (blue). **(F)** Analyzed 12 min tracks of each particle, indicated by blue lines. **(G)** Analyzed 2 h track of the single particle indicated by a blue arrow in **(F)**. The scale bar indicates 200 μm.

The particle analysis using PKA3 was performed on each frame of the time-lapse phase-contrast microscopic images (Figure 1). Particles were defined as a group of pixels that could be regarded as one connection on the image (Figure 1D). Detailed definitions of particles are described in the analysis overview in Supplementary Materials. Single amoeba, a cluster of multiple amoebae, and debris may be recognized as particles. To detect particles, edge detection using a gradient of intensity was used. In the phase-contrast microscopic images that contained the halo and shade-off artifacts, the edges did not surround the particles singly but in a complicated, overlapping pattern (Figure 1B). Although the edge data were not sufficient to accurately capture the shape of the particles, the approximate shape, position, size, and number of particles could be captured. Pixels were identified as particles, noise, or halos according to their intensity (Figure 1C, analysis overview in Supplementary Materials).

**Figure 2:**
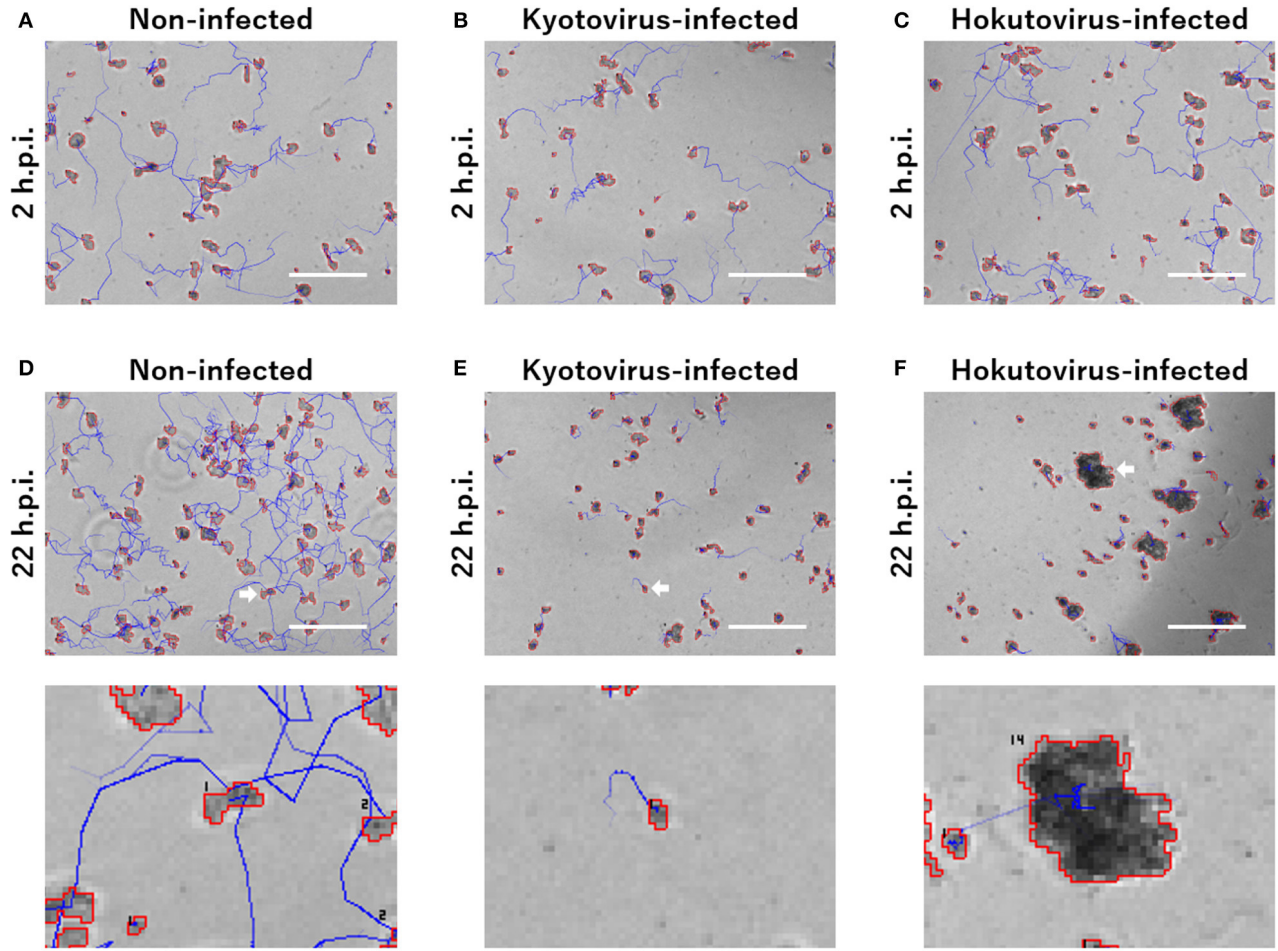
Merged images of phase-contrast microscopy and particle and tracking analysis. **(A–F)** Areas outlined with red lines indicate the particles, and blue lines indicate the track of each particle. Each image of **(A–C)** and upper **(D–F)** is a 240 × 180 pixels area cut out from the original image. The scale bar indicates 200 μm. Lower images of **(D–F)** are enlarged images of the cell indicated by white arrows in the upper images. The numbers in the lower images are the multiplicity.

For the particle tracking analysis in PKA3, one particle in a frame was simply defined as moving to the nearest particle in the next frame. According to this definition, the time-lapse intervals needed to be short enough to allow this analysis. In the time-lapse images of 1 frame per minute of non-infected amoebae, the particles of one frame (outlined in red in Figure 1E) largely overlapped with the particles of the next frame (outlined in blue in Figure 1E). The 10 m tracks of each particle and the 2-h track of single particles that were outputted in this way, are shown in Figures 1F,G, respectively. This analysis of time-lapse images can output the migration distance and direction of the particles. In addition, normal amoeba cells split by cell division, or sometimes adhere to each other and behave as a single particle. Analyzing the multiplicity, that is the putative number of cells adhered in a single particle, can be used to identify these amoeba-specific behaviors. The multiplicity was simply added when the particles adhered to each other and was shared depending on the size of the particles when they split. On the contrary, when a particle with a multiplicity of 1 divided, it resulted in two particles that were both considered to have a multiplicity of 1. By combining these analyses, images like Figure 2 were output for each frame. The results of these image analyses can be output as time-series changes in estimated cell number, average particle shape, size, migration distance, and direction. As shown in Figure 2D, particles with long tracks, indicated by blue lines, were observed in non-infected amoebae. In kyotovirus-infected amoebae, small particles with short tracks were observed at 22 h post-infection (h.p.i.) (Figure 2E). In hokutovirus-infected amoebae, large particles that were thought to be aggregates of multiple cells, as expected from the multiplicity, were observed (Figure 2F).

### Particle and Tracking Analysis of Amoebae

Time-lapse capture of phase-contrast microscopic images was continued for 36 h, resulting in a 960 × 720 pixels image of 2,880 frames per sample. The number of non-infected amoeba cells gradually increased during the time-lapse capture. However, the number of kyotovirus-infected amoeba cells did not appear to increase. Small and rounded amoebae, considered to show CPE, were observed at 36 h.p.i. (Supplementary Figure 1) when the amoebae were infected with kyotovirus. Hokutovirus-infected amoeba cells formed “bunches” at 24 h.p.i (Supplementary Movie 1). The “bunches” dissolved at 36 h.p.i., and instead, small and rounded amoebae were observed.

**Figure 3:**
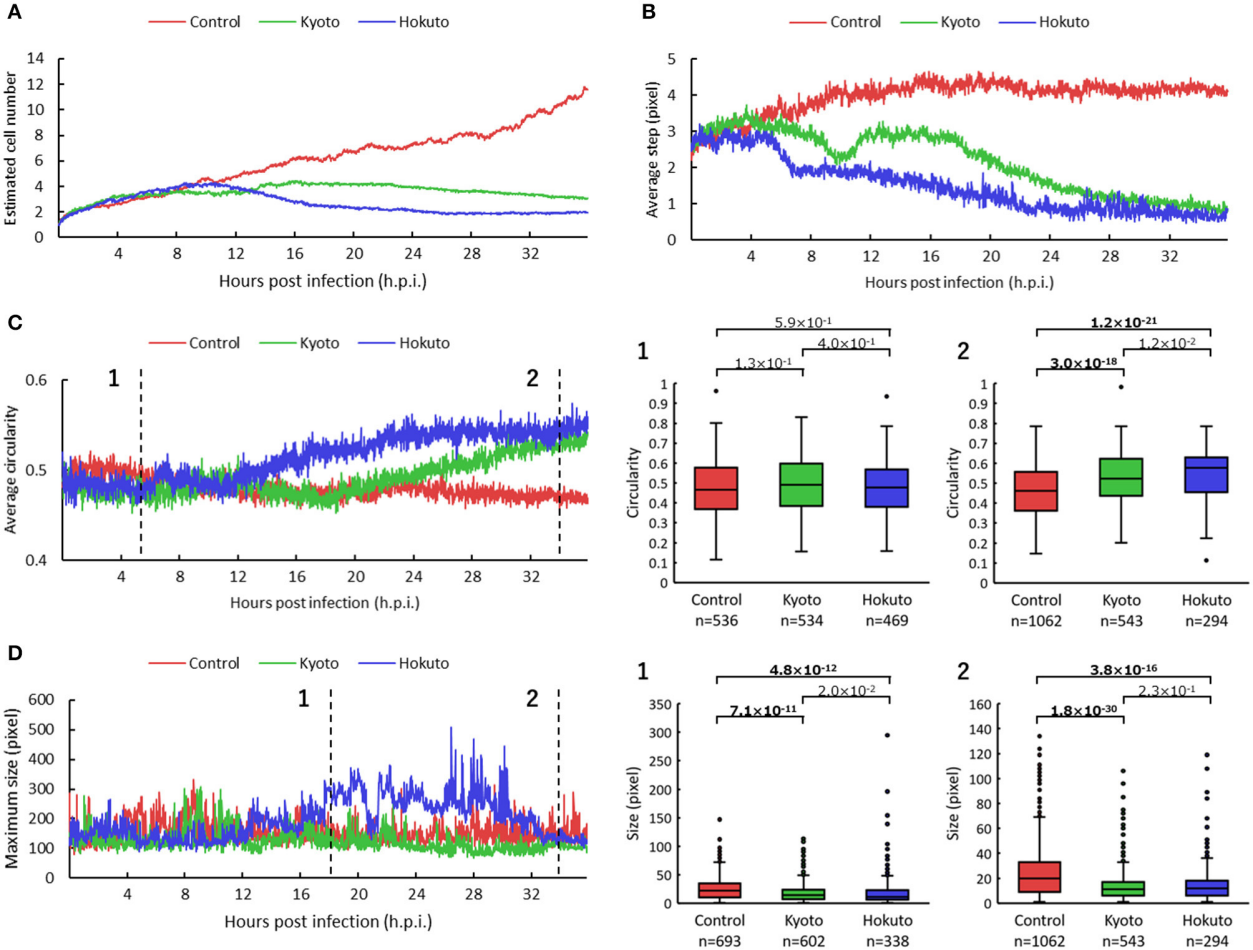
Comparison of particle and tracking analyses among non-infected and kyotovirus- or hokutovirus-infected amoeba cells. **(A–D)** Time-series change in estimated cell number **(A)**, average step **(B)**, circularity **(C)**, maximum size **(D)** of the particles. “Control,” “Kyoto,” and “Hokuto” indicate non-, kyotovirus- and hokutovirus- infected amoebae, respectively. The vertical axis of **(A)** represents the magnification when compared with 0 h.p.i. for each sample. The boxplots of **(C,D)** show values of all particles for two time points, respectively. *n* indicates the number of particles contained in each result. Values above the boxplots correspond to *p*-values of Mann–Whitney U two-sided tests; bold letters indicate significant differences (*p* < 0.01).

For kinetic analysis of these amoeba cells, we performed particle and tracking analysis (Supplementary Movies 2–4). The estimated number of cells was obtained by the sum of the multiplicity of the particles for each frame. The estimated number of non-infected amoeba cells, revealed by particle and tracking analysis, continued to increase up to 36 h.p.i. (Figure 3A), consistent with that shown in original phase-contrast microscopic images (Supplementary Figure 1). On the other hand, in the case of amoebae infected with kyotovirus or hokutovirus, the number of cells increased in the same way as that in non-infected amoebae during the early period post-infection, but it remained flat or gradually decreased from 12 h.p.i. (Figure 3A). These results suggest that the changes in cell numbers over time could be an indicator that quantitatively and clearly shows that the amoeba cells were being externally affected.

The results for particle movement were obtained from the average “step,” which is the length connecting the center point of each particle of one frame with that of the next frame. The average step at about 12 h.p.i. was markedly shorter in kyotovirus- or hokutovirus-infected amoeba cells than in non-infected amoebae (Figure 3B), although there were no intuitive differences between virus-infected and non-infected amoebae by simple observation of microscopic images at 12 h.p.i. (Supplementary Figure 1). These results suggest that the analysis of the steps of the particles can detect the infection of amoeba cells with these viruses earlier than that by conventional observation of CPE and can be useful in the detection of infection with other viruses. Interestingly, kyotovirus-infected amoeba cells once shortened their steps and then temporarily lengthened them again at about 10 h.p.i (Figure 3B). This phenomenon was also observed in another sample (Supplementary Figure 2). This hitherto unknown kinetics of kyotovirus-infected amoeba cells was revealed in the current study.

The shape of cells was quantified using the average “circularity” of particles in each frame. The circularity of each particle was calculated by the equation: 4 π S/L^2^ (Cox, 1927). S is the area of the particle, π is the circumference ratio, and L is the perimeter of the particle. Kyotovirus- or hokutovirus-infected amoeba cells gradually increased in average circularity (Figure 3C). At 34 h.p.i., the overall circularity of virus-infected amoeba cells was significantly higher than that of non-infected amoebae (boxplot of Figure 3C). The infection of amoeba cells with kyotovirus or hokutovirus could be distinguished by determining maximum particle size. Hokutovirus-infected amoeba cells increased to a maximum particle size between 12 and 30 h.p.i., probably because of the formation of “bunches” (Figure 3D). On the other hand, at 34 h.p.i., the overall particle sizes of amoebae infected with either virus were significantly smaller than those of the non-infected amoebae (boxplot of Figure 3D). These results suggest that the overall circularity and particle size can be useful to quantitatively identify the amoeba cells showing CPE or virus infection.

### Finding Bias of Amoebae Movement

**Figure 4:**
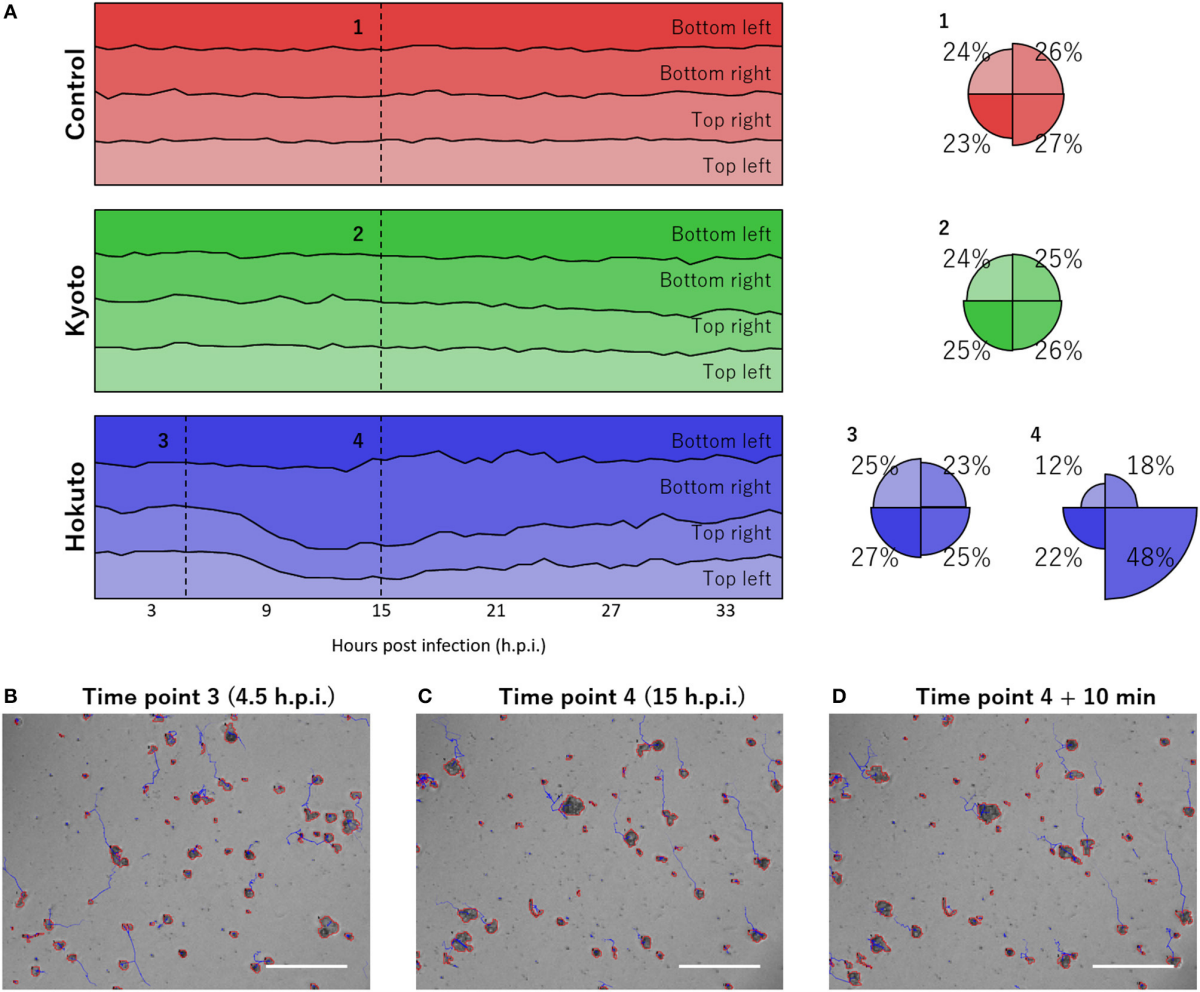
Bias in the directions of particle movement. **(A)** Stacked line charts of percentage of directions of particle movement in the last hour. “Control,” “Kyoto,” and “Hokuto” indicate non-, kyotovirus-, and hokutovirus- infected amoebae, respectively. Circles indicate the value at numbered time points. **(B–D)** Phase-contrast microscopic images at time point 3 **(B)**, time point 4 **(C)**, and 10 min after time point 4 **(D)**. The scale bar indicates 200 μm.

Notably, using the tracking analysis of PKA3, it was observed that many particles in hokutovirus-infected amoebae moved in the same direction. This phenomenon was quantitatively found by dividing the moving direction into four quadrants and counting particles that moved to each direction (Figure 4A). When the bias is highest, 48% of all particles moved in the same direction if infected with hokutovirus, and 31% if non-infected or kyotovirus-infected. Although some of the tracks continued randomly from the particles in the analysis images of 4 h.p.i. (Figure 4B), almost all the tracks from the particles in the analysis images of 15 h.p.i. continued to the top left (Figures 4C,D). This bias in amoebae movement was also visually observed (10–20 s of Supplementary Movies 1, 4) and lasted ~10 h. This phenomenon preceded the increase in the size of the particles, suggesting that this movement is a functional process involved in the formation of the “bunches” of hokutovirus-infected amoeba cells.

## Discussion

**Figure 5:**
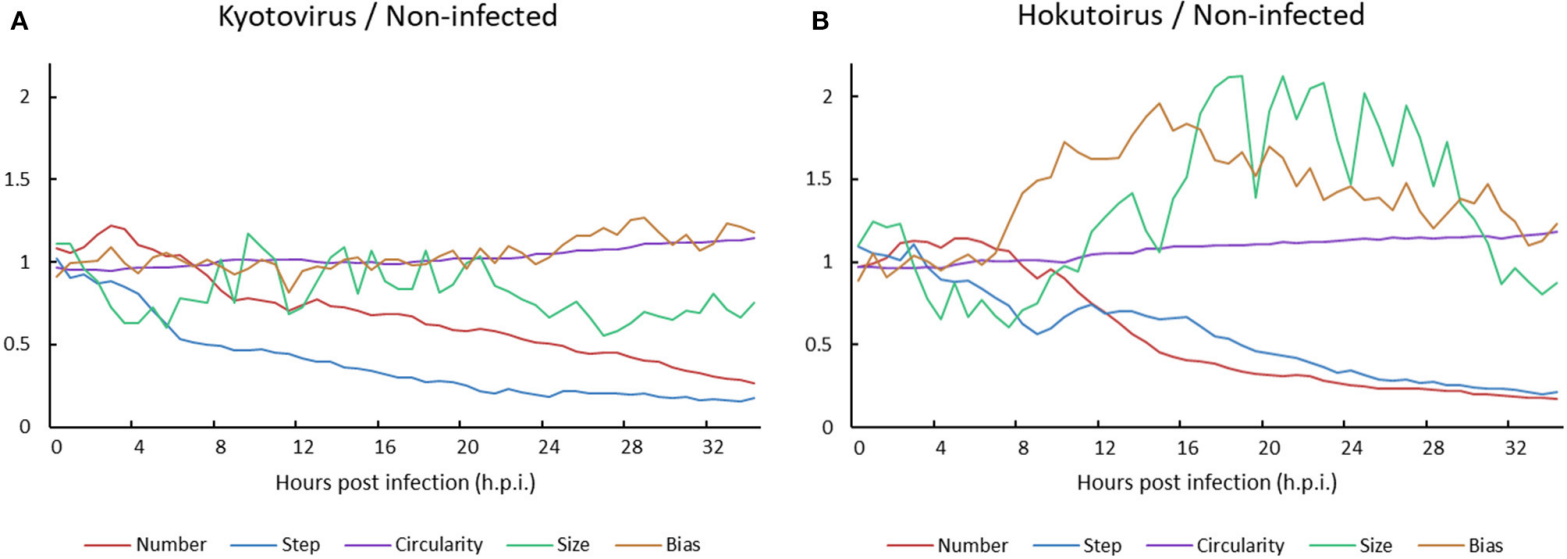
Ratio of **(A)** kyotovirus-infected or **(B)** hokutovirus-infected amoeba cells to non-infected amoeba cells.

A comparison of kyotovirus- or hokutovirus-infected amoeba with non-infected amoeba revealed the time-dependent kinetic behavior of each amoeba cell. Until approximately 8 h.p.i., the particle size and steps of kyotovirus- or hokutovirus-infected amoeba cells decreased. Subsequently, the ratio of virus-infected to non-infected amoeba cell number decreased (Figure 5). It may be largely a result of the increased number of non-infected amoeba cells relative to infected amoeba cells. The circularity of virus-infected amoebae gradually increased, which is consistent with the increased circularity in *Acanthamoeba* CPE caused by mimivirus infection (Yaakov et al., 2019). At ~10 h.p.i., hokutovirus-infected amoebae showed an increase in the direction bias of particles, and maximum particle sizes rapidly increased because of the formation of a “bunch” (Figure 5B). These results suggest that the sequential observations of virus-infected amoebae using time-lapse images clearly reveal the previously unknown ethological behaviors of amoeba cells and the differences between non-infected and virus-infected amoebae.

In this study, we investigated two fundamental and important topics. One was the development of a kinetic analysis program of moving cells using phase-contrast microscopic images, PKA3. Image analysis, especially kinetic analysis of moving cells, of phase-contrast microscopic images is rarely performed because of the presence of “halo” and “shade-off” artifacts in the process of phase-contrast, as described above. This study showed that even if the image analytic algorithm is simple, useful information can be obtained from phase-contrast microscopic images by combining algorithms specialized for this purpose. Customizing these algorithms to suit experimental conditions makes it easier to analyze moving cells in time-lapse phase-contrast microscopic images. Image analysis based on a common and versatile phase-contrast microscope, could provide useful information in various biological studies, mainly using the analysis of tracking cell migration.

Another important topic that we investigated was the revealing behaviors of amoebae with or without virus infection. We detected changes in the number of cells and the appearance of CPE in marseillevirus-infected *Acanthamoeba* quantitatively and earlier than that by conventional observations. These contiguous and quantitative data obtained by time-lapse capture of phase-contrast microscopic images should unravel the time and order of the response of amoebae to external effects such as viruses and will provide useful information for the study of the life cycle of amoebae and viruses. This analysis can detect the “abnormal” behaviors of amoebae without relying on human eyes, such as the time-course of “bunch” formation by hokutovirus, decrease in movement of virus-infected amoebae, and the direction bias of hokutovirus-infected amoebae shown in this study. The analysis also showed that hokutovirus-infected amoeba cells behaved in a collective manner, which may be involved in the formation of “bunches.” It suggests that the amoebae formed “bunches” not because of normal movement but to assemble in response to some unknown signals, which caused the direction bias. This phenomenon may be an antiviral strategy of the host, similar to Faustovirus-infected amoeba releasing a factor that triggers the encystment of neighbor cells (Borges et al., 2019). Or it may be a viral spreading strategy, similar to the efficient spread of HIV-1 through contact and fusion of host cells (Bracq et al., 2017). Studying the source of the direction bias and the conditions of amoeba cells inside the “bunch” may be helpful to understand the viral infection.

Analyzing a large number of samples could also lead to the discovery of unknown behaviors of various types of cells, including unicellular organisms and individual cells of multicellular organisms. Additionally, it will promote the detection of unknown giant viruses that affect amoeba behavior. Amoeba cells show various types of “abnormal” behaviors when they are infected with various giant viruses. For example, mimivirus-infected amoeba cells show drastically decreased cell motility and death by the “explosion” of proliferated mimivirus particles (Akashi and Takemura, 2019). On the other hand, medusavirus-infected amoeba cells show moderately decreased cell motility later than those of mimivirus-infected cells and change the cells to a cyst-like formation (Yoshikawa et al., 2019). Kinetic analytical program PKA3, developed in this study, could reveal these CPEs of amoeba quantitatively and expand the study of virus-host interactions from the viewpoint of the host and its behavior. Furthermore, this analytical program can be applied to other cells, including cancer cells, lymphocytes, macrophages, and neurons, for elucidation of the physiological mechanisms of cancer metastasis, function of the immune response, and the structure of the neural network.

## Data Availability Statement

All datasets generated for this study are included in the article/Supplementary Material.

## Author Contributions

SF, KA, and MT designed the research. SF developed the kinetic analysis program, PKA3, and performed particle and tracking analyses. KA and MK performed the culturing of amoebae, infection of cultured amoebae cells with viruses, and the capture of time-lapse phase-contrast microscopic images. SF and MT wrote the initial manuscript. All authors contributed to the finalization of the manuscript.

### Conflict of Interest

The authors declare that the research was conducted in the absence of any commercial or financial relationships that could be construed as a potential conflict of interest.
