# Supplementary material for: Kinetic Analysis of the Motility of Giant Virus-Infected Amoebae Using Phase-Contrast Microscopic Images
Source: Front Microbiol. 2020 Jan 17;10:3014. doi: 10.3389/fmicb.2019.03014 (PMC6988830; doi:10.3389/fmicb.2019.03014)
Supplement: Supplementary file 1 [file Data_Sheet_1.docx]

Supplementary Material

Kinetic analysis of the motility of giant virus-infected amoebae using phase-contrast microscopic images

Sho Fukaya^1^*, Keita Aoki^1^, Mio Kobayashi^2^ & Masaharu Takemura^1,2^*

**Affiliations:**

^1^Laboratory of Biology Education, Department of Mathematics and Science Education, Graduate School of Science, Tokyo University of Science, Shinjuku, Tokyo, Japan

^2^Laboratory of Biology, Department of Liberal Arts, Faculty of Science, Tokyo University of Science, Shinjuku, Tokyo, Japan

***Corresponding authors:** 1717706@ed.tus.ac.jp **(SF),** giantvirus@rs.tus.ac.jp **(MT)**

1. **Supplementary Figures**

**Supplementary Figure S1**

**Supplementary Figure S2**

1. **Supplementary Movies**

**Supplementary Movie S1**

**Supplementary Movie S2**

**Supplementary Movie S3**

**Supplementary Movie S4**

1. **Supplementary Method**

**3.1 Overview of analysis**

# Supplementary Figures


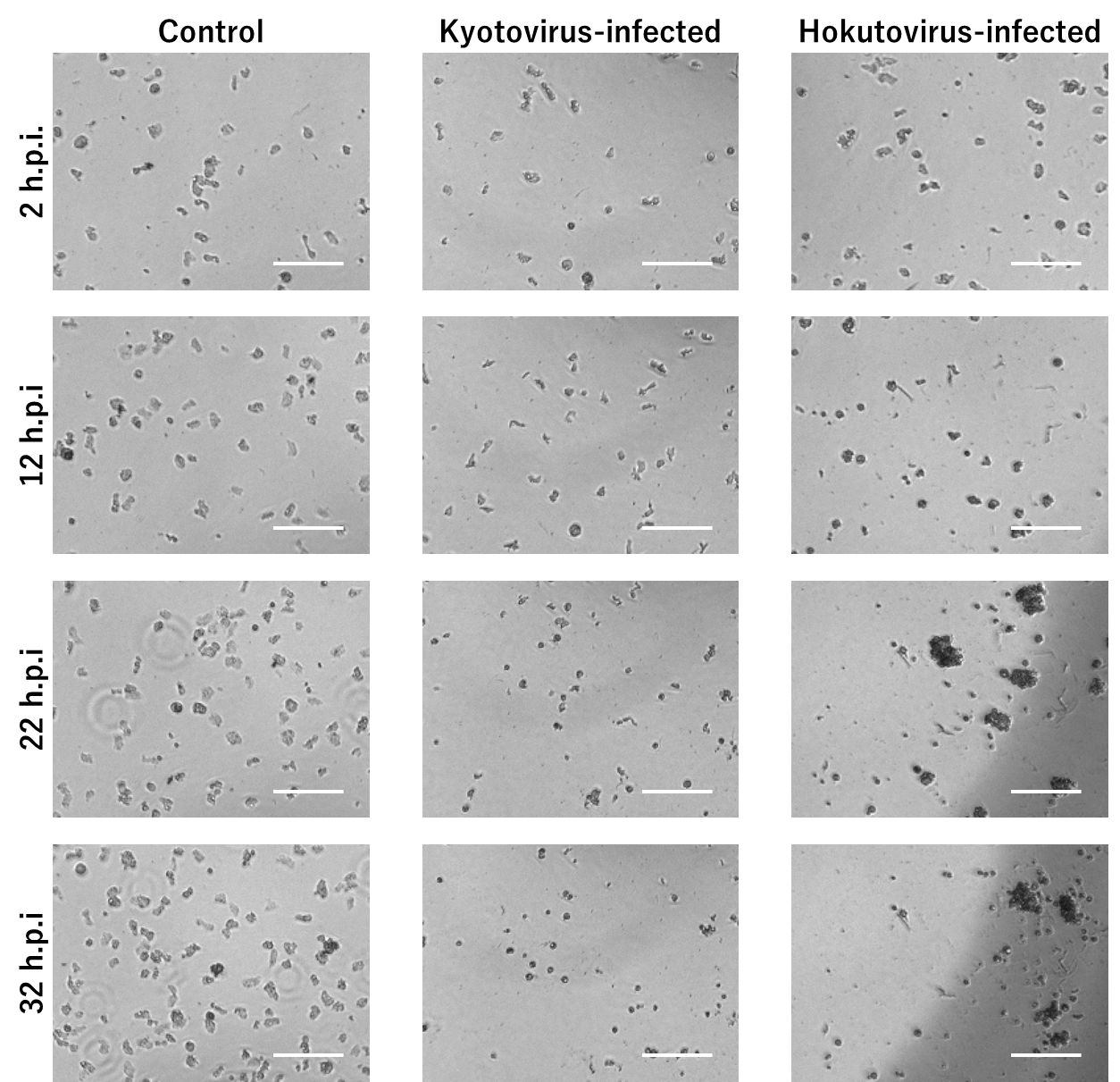


**Supplementary Figure S1.** Comparison between the particle sizes of non-infected and virus-infected amoeba cells. Amoeba cells were cultured in PYG medium and grown on 96-well microplates with 100 μL PYG medium in each well, followed by infection of kyotovirus or hokutovirus (M.O.I = 1). These images were obtained by time-lapse capture of phase-contrast microscopic images. The scale bar indicates 200 μm.


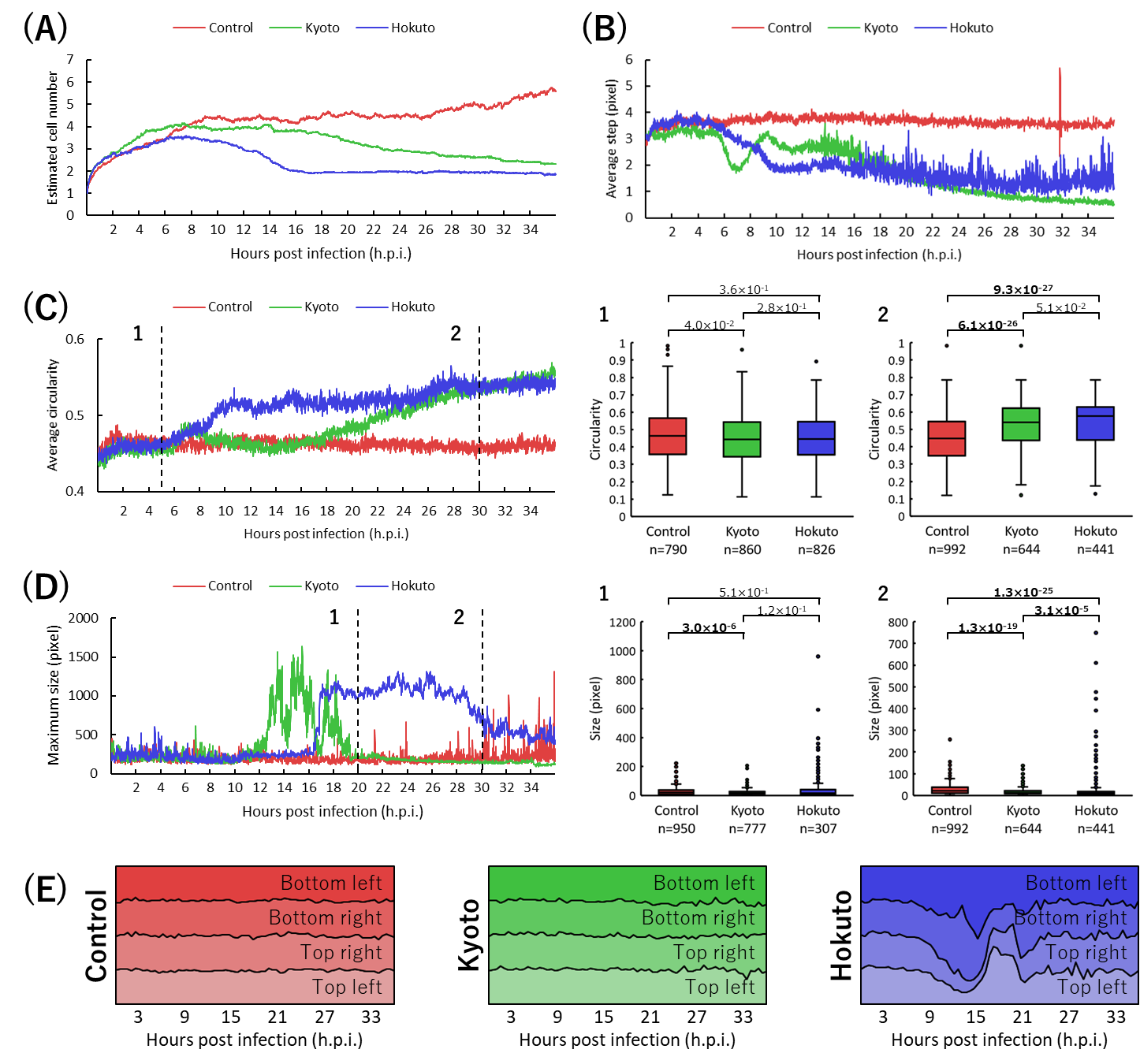


**Supplementary Figure S2.** Particle and tracking analyses of other samples of infected amoebae. **(A)-(E)** Time-series change in estimated cell number. **(A)** average step, **(B)** circularity, **(C)** maximum size, **(D)** of the particles, and bias in the directions of particle movement, **(E)** ‘Control’, ‘Kyoto’, and ‘Hokuto’ indicate non-, kyotovirus-, and hokutovirus-infected amoebae, respectively. The vertical axis of **(A)** represents the magnification when compared with 0 h.p.i. for each sample. The boxplots of **(C)** and **(D)** show values of all particles for two time points, respectively. n indicates the number of particles contained in each result. Values above the boxplots indicate p-values of Mann–Whitney U two-sided tests; bold letters indicate significant differences (*p* < 0.01).

# Supplementary Movies

**Supplementary Movie S1.** Original movie of hokutovirus-infected amoeba cells. Amoeba cells were cultured on a 96-well microplate in 100 μL PYG medium, and infected with hokutovirus. Time-lapse images were captured as described in the methods section.

**Supplementary Movie S2.** Particle and tracking analysis of non-infected amoeba cells. Amoeba cells were cultured on a 96-well microplate in 100 μL PYG medium. Time-lapse images were captured as described in the methods section. Particle and tracking analyses were performed by PKA3.

**Supplementary Movie S3.** Particle and tracking analysis of kyotovirus-infected amoeba cells. Amoeba cells were cultured on a 96-well microplate in 100 μL PYG medium, and infected with kyotovirus. Time-lapse images were captured as described in the methods section. Particle and tracking analyses were performed by PKA3.

**Supplementary Movie S4.** Particle and tracking analysis of hokutovirus-infected amoeba cells. Amoeba cells were cultured on a 96-well microplate in 100 μL PYG medium, and infected with hokutovirus. Time-lapse images were captured as described in the methods section. Particle and tracking analyses were performed by PKA3.

# Supplementary Method

## Overview of analysis

In the analysis using PKA3, the edge was found in the region where the intensity was different from the surrounding pixels, and the larger the difference, the clearer the edge was. In the phase-contrast microscopic images, the cells appeared as areas with pixels of different intensity compared to the surroundings, and thus, cells were analyzed so as to be surrounded by edges. Because of artifacts such as halos, the intensity increased and decreased around the cells. Edges were also observed inside the cells owing to vacuoles and other structures. Therefore, cells were surrounded by various edges. By connecting edges in order of clarity, the image was divided into a number of areas surrounded by small edges (herein referred to as proto-particles) and an area not surrounded by edges. Proto-particles were then labeled peaks of intensity, valleys of intensity, or neither of these. The program identified proto-particles of peaks as halos, of valleys as particle-candidates, and neither of these as noise. Particles in this study were obtained by combining proto-particles from adjacent particle-candidates and integrating pixels left behind inside the combined particle-candidates. Finally, particles with less than 8 pixels were removed because they were too small to be cells.
